# Computational prediction of type III secreted proteins from gram-negative bacteria

**DOI:** 10.1186/1471-2105-11-S1-S47

**Published:** 2010-01-18

**Authors:** Yang Yang, Jiayuan Zhao, Robyn L Morgan, Wenbo Ma, Tao Jiang

**Affiliations:** 1 Department of Computer Science and Engineering, Information Engineering College, Shanghai Maritime University, 1550 Haigang Ave., Shanghai 201306, PR China; 2 Ministry of Education Key Laboratory for Biodiversity Science and Ecological Engineering, School of Life Sciences, Fudan University, 220 Handan Road, Shanghai, 200433, PR China; 3 Department of Plant Pathology and Microbiology, University of California, Riverside, CA 92521, USA; 4 Department of Computer Science and Engineering, University of California, Riverside, CA 92521, USA; 5 Institute for Integrative Genome Biology, University of California, Riverside, CA 92521, USA; 6 College of Information Science and Technology, Tsinghua University, Beijing 100084, PR China

## Abstract

**Background:**

Type III secretion system (T3SS) is a specialized protein delivery system in gram-negative bacteria that injects proteins (called effectors) directly into the eukaryotic host cytosol and facilitates bacterial infection. For many plant and animal pathogens, T3SS is indispensable for disease development. Recently, T3SS has also been found in rhizobia and plays a crucial role in the nodulation process. Although a great deal of efforts have been done to understand type III secretion, the precise mechanism underlying the secretion and translocation process has not been fully understood. In particular, defined secretion and translocation signals enabling the secretion have not been identified from the type III secreted effectors (T3SEs), which makes the identification of these important virulence factors notoriously challenging. The availability of a large number of sequenced genomes for plant and animal-associated bacteria demands the development of efficient and effective prediction methods for the identification of T3SEs using bioinformatics approaches.

**Results:**

We have developed a machine learning method based on the N-terminal amino acid sequences to predict novel type III effectors in the plant pathogen *Pseudomonas syringae *and the microsymbiont rhizobia. The extracted features used in the learning model (or classifier) include amino acid composition, secondary structure and solvent accessibility information. The method achieved a precision of over 90% on *P. syringae *in a cross validation study. In combination with a promoter screen for the type III specific promoters, this classifier trained on the *P. syringae *data was applied to predict novel T3SEs from the genomic sequences of four rhizobial strains. This application resulted in 57 candidate type III secreted proteins, 17 of which are confirmed effectors.

**Conclusion:**

Our experimental results demonstrate that the machine learning method based on N-terminal amino acid sequences combined with a promoter screen could prove to be a very effective computational approach for predicting novel type III effectors in gram-negative bacteria. Our method and data are available to the public upon request.

## Background

Protein secretion is an essential mechanism for bacterial survival in their surrounding environment. Gram-negative bacteria have two membranes, the outer membrane and the inner membrane. Therefore, their secretion systems are more complex compared to gram-positive bacteria. Up to now, researchers have discovered six specialized secretion systems in gram-negative bacteria. Among them, the type III secretion system (T3SS) is indispensable for the pathogenesis of a large variety of plant and animal pathogens, many of which are responsible for the most devastating diseases. For example, T3SS has been identified from the animal pathogens *Salmonella*, *Yersinia*, *Shigella *and *Escherichia*, and plant pathogens *Pseudomonas*, *Erwinia*, *Xanthomonas*, *Ralstonia*, *Pantoea*, *etc*. [1,2]. Using T3SS, these pathogens inject virulence proteins, so-called type III effectors (T3SEs), directly into the host cells. Once inside, T3SEs target their specific host substrates and promote disease development. Recently, T3SS and T3SEs are also found in non-pathogenic bacteria. Specifically, T3SS is important for some microsymbiont rhizobia to infect legumes during nodulation [3].

**Figure 1:**
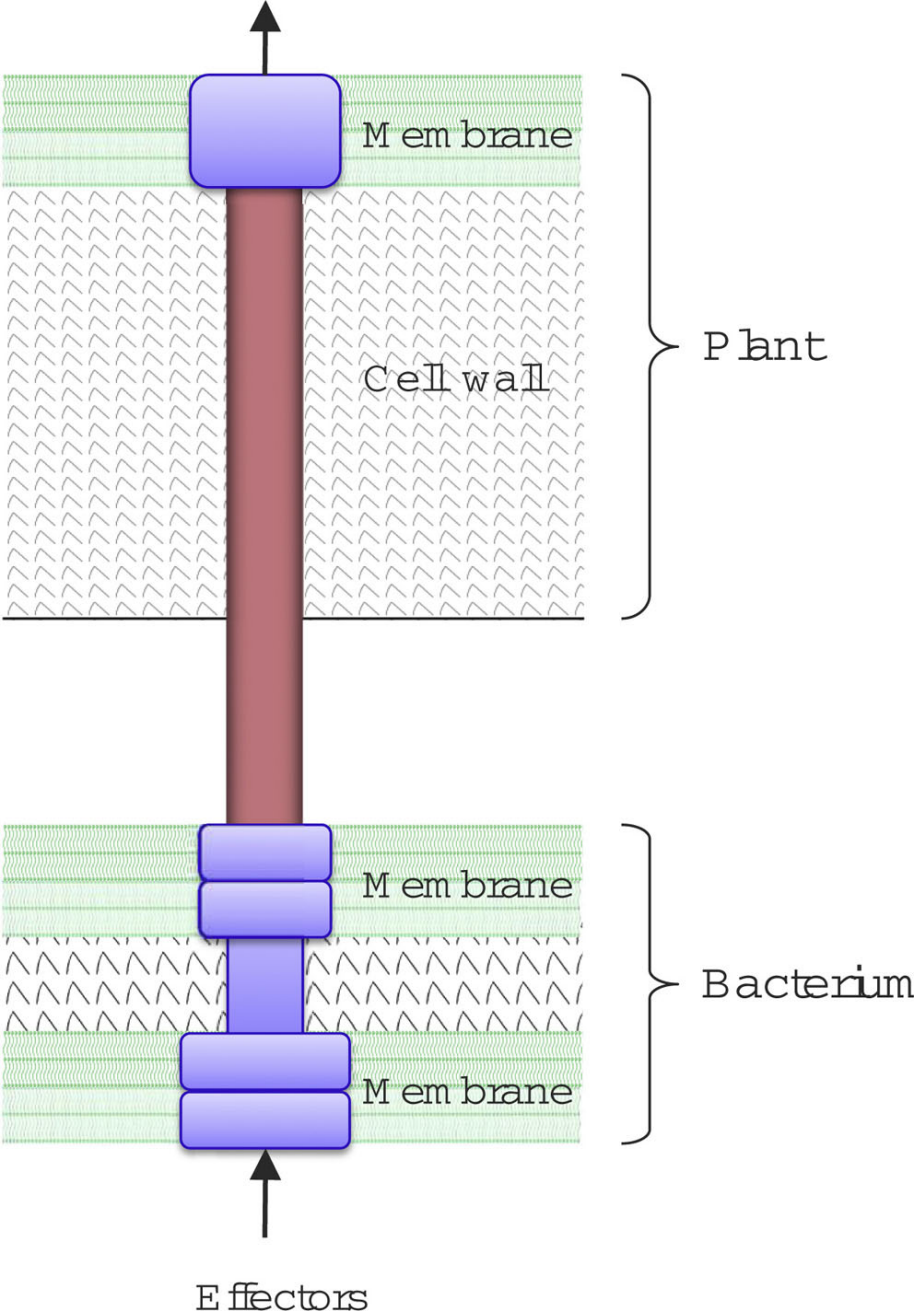
**The T3SS apparatus in Pseudomonas syringae**.

The structural components of T3SS from different bacteria are highly conserved. A typical type III machinery includes a needle and bases embedded in the inner and outer bacterial membranes as shown in Fig. 1. The needle, spanning the cell membranes of both the bacterium and the host, is a channel for delivering effectors into the host cytoplasm. Unlike the apparatus proteins, type III secreted proteins are highly variable even among different strains of the same bacterial species. This is mainly because they evolve fast in order to adapt to different hosts and respond to the resistance from the host immune systems [4].

**Figure 2:**
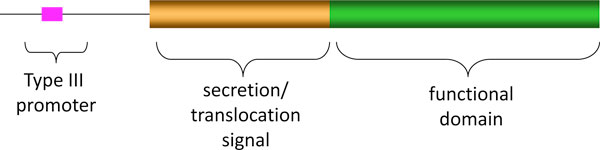
**The composition of a typical effector protein**.

Because of the essential biological functions of T3SEs during bacterial association with eukaryotic hosts, a lot of research has been conducted to identify effector proteins. A major challenge for the identification of T3SEs is that there is no defined signal peptide or motif discovered from the amino acid sequences of known effectors. Therefore, it is notoriously difficult to predict novel T3SEs using bioinformatic approaches. The plant pathogen *Pseudomonas syringae *has been a model for the research of type III effectors. Thus far, over two hundred T3SEs have been identified and confirmed in *P. syringae *strains, more than the total number of effectors identified from all other bacterial species. Therefore, we conjecture that a large portion of T3SEs in other bacteria remain unknown. In *P. syringae*, the commonly used method for identifying T3SEs is functional screen [5,6], in which a known T3SE (*i.e*., AvrRpt2) is used as a marker for type III-dependent translocation. These functional screens are based on the modular character of type III effectors. A typical type III effector usually contains a secretion/translocation signal in the N-terminus and a functional domain in the C-terminus (Fig. 2). In a functional screen, the candidate protein (its N-terminus or full length) is fused to the functional domain of AvrRpt2. If the candidate protein has the secretion and translocation signal, it would direct the translocation of the functional domain of AvrRpt2, which would then result in a hypersensitive response in *Arabidopsis thaliana*. This method is accurate, but very laborious and time-consuming especially when it has to meet the increasing need of whole-genome screens.

As the sequencing techniques gained breakthrough for the past decade, the complete genomic sequences of many bacteria utilizing T3SS are available. Therefore, bioinformatics approaches for predicting T3SEs based on genomic data have attracted a great deal of research interests [7,8]. There are three main computational methods: promoter-based, secretion/translocation signal-based and homology-based, that could be used for the prediction. However, none of these methods have satisfactory performance.

In a bacterium, genes encoding the T3SS apparatus and T3SEs usually have a conserved regulatory motif in their promoters [9], as shown in Fig. 2. For example, *P. syringae *has a motif called the *hrp *box that has the concatenated sequence pattern GGAACC-N(14-17)-CCACNNA [10-12]. Here, an N means any nucleotide and "N(14-17)" represents a sequence of 14 to 17 nucleotides. Vencato *et al*. [12] built an HMM trained on *P. syringae *pv. tomato strain DC3000 to scan the genome of *P. syringae *pv. phaseolicola strain 1448A. They obtained a total of 44 high-probability candidate *hrp *promoters on the chromosome and two endogenous plasmids. Although the promoter search is an efficient method for identifying effectors, it has several limitations. (a) Not all genes preceded by the *hrp *promoters encode effectors. (b) Some effector genes may not have a *hrp *promoter. Therefore, the *hrp *promoters cannot be used as a necessary condition. (c) The *hrp *boxes contained in some of the effector gene promoters are rather weak motif instances (*i.e*., they actually deviate from the consensus pattern quite a bit). This makes the determination of a true *hrp *promoter very difficult.

Researchers have been trying to detect amino acid composition biases in T3SEs, especially in the N-termini. Generally speaking, the first 15 amino acids are most essential and the first 50 amino acids are usually sufficient for secretion. However, some researchers argue that maximal secretion or translocation requires the first 100 amino acids [13-15]. Although not much sequence similarity could be observed in the known T3SE sequences, Guttman *et al*. [5] reported that the first 50 amino acids of *P. syringae *effectors had a high proportion of Ser and a low proportion of Asp residues. Similarly, Petnicki-Ocwieja *et al*. [16] identified specific biophysical features of the first 50 amino acids of effector proteins in *P. syringae *pv. tomato: (a) the presence of solvent-exposed amino acids in the first five amino acids, (b) the lack of Asp or Glu residues in the first 12 amino acids, and (c) the amphipathicity of the first 50 amino acids. Again, these observations only revealed some statistical biases in the N-terminal region instead of providing specific sequences responsible for the protein secretion. Moreover, many effectors do not fulfill these requirements. Some effectors even possess none of these features [8]. Petnicki-Ocwieja *et al*. [16] mentioned that although their attempts to discover motifs in the first 50 aa of these proteins using known programs failed, several patterns emerged when these amino acid residues were examined based on their biophysical properties and solvent-exposed substitutability.

Aligning candidates with known effectors for homology search would be the most straightforward way to identify T3SEs. However, T3SEs have great sequence diversity through fast evolution and many T3SEs have no homology with any protein in the public databases. Moreover, this method will not lead to the identification of novel effectors.

In this study, our goal is to predict novel type III secreted proteins from genomic data. We have developed a machine learning method based on amino acid sequences. We used amino acid composition, secondary structure and solvent accessibility information to represent protein sequence features, and adopted the support vector machine (SVM) to classify feature vectors as effectors and non-effectors. The method was tested on the *P. syringae *data set through 5-fold cross validation. We then applied this method to a rhizobia data set. Combined with promoter search, we predicted 57 candidates from four rhizobial strains, in which 17 are confirmed T3SEs by wet-bench experiments.

## Results and discussion

### Data source

Compared with other bacteria, *Pseudomonas syringae *has been used as a model organism in the study of T3SEs, with the highest number of effectors identified. Therefore, we collected training data from this species. The positive data set consists of all the 283 confirmed effectors from *P. syringae *pv. tomato strain DC3000, *P. syringae *pv. syringae strain B728a and *P. syringae *pv. phaseolicola strain 1448A. We also constructed a non-redundant subset, in which the homologous effectors with sequence similarity higher than 60% were excluded (Table 1). The negative data set was extracted from the genome of *P. syringae *pv. tomato strain DC3000. We eliminated all the proteins related to T3SS, as well as the hypothetical proteins. The remaining proteins constitute the non-effector data set. It should be noted that the negative data set may contain some unknown effectors, and thus the negative data could be overestimated. Table 1 shows that the class distribution is very imbalanced, *i.e*., the positive data size is much smaller than the negative data size. Moreover, the class distribution becomes even more imbalanced after removing the redundancy since many of the known effectors were identified via homology search.

**Table 1:** Positive and negative sample numbers in the two data sets. Set I is the redundant data set and set II the non-redundant data set.

Data	Positive	Negative	Total
I	283	3779	4062
II	108	3424	3532

### Experimental settings and evaluation criteria

We used the SVM as our classifier, and performed a 5-fold cross validation test and grid search on the training data to find the optimum (SVM) parameters. All computation tasks were conducted on a Pentium IV desktop PC with dual CPU (2.8 GHz) and 2 GB RAM.

Multiple measures were used to assess the performance of our proposed method, including precision (*P*), recall (*R*) and total accuracy (*TA*). The former two measures are used to measure the prediction quality of effectors, and *TA *is used to measure the overall prediction quality. The precision and recall can be defined in terms of the number of true positives (*TP*), the number of false positives (*FP*), and the number of false negatives (*FN*) as follows. We define(1)(2)

where *P *is the ratio of the samples correctly classified into the positive class compared to the total number of samples classified into the positive class, and *R *is the ratio of the samples correctly classified into the positive class compared to the number of known effectors. TA is the ratio of the samples classified correctly compared to the total size of the data set.

### Feature vectors

To represent protein sequence features, we considered three feature extraction methods: (a) amino acid composition (AAC), (b) *k*-mer composition, and (c) amino acid composition in terms of their different secondary structures and solvent accessibility states, called the SSE-ACC method.

The AAC method converts a protein sequence into a 20-dimensional feature vector, recording the composition or frequency of each of the 20 amino acids. This method is the easiest to implement but has the most information loss. The *k*-mer method retains the amino acid ordering and neighborhood information up to the length of each *k*-mer (*i.e*., *k*). However, as *k *increases, this method introduces a 20^*k*^-dimensional feature space, which is computationally intractable. On the *P. syringae *data set, the *k*-mer method did not show an obvious advantage over AAC, and its performance showed very little improvement when *k *was increased.

The SSE-AAC method generates 100-dimensional feature vectors. The first 60 dimensions are used to describe the frequency of each amino acid in each of the three possible secondary structure elements, *i.e*., strand (E), helix (H) and coil (C). The value of each dimension is calculated by(3)

where *j *= {H, E, C},  is the frequency of amino acid *i *in secondary structure element *j*, and *L *is the length of the sequence. We used N-terminal sequences in stead of full-length sequences. As mentioned before, the maximal secretion or translocation may require the first 100 amino acids [13-15]. Therefore, in our experiments, the first 100 amino acids were used.

The last 40 dimensions represent the frequency of each amino acid (among the first 100 N-terminal residues) having each of the two possible solvent accessibility states, namely buried (B) and exposed (E), and are calculated similarly as Eq. 3, with *j *= {B, E}. These two types of information are combined into a single feature vector called SSE-ACC.

Amino acid frequencies on different secondary structure elements and solvent accessibility states were first used in protein fold classification by Shamim *et al*. [17]. This method was also demonstrated to be effective in our experiments. We have carried out some comparisons on different feature vectors. Our results show that the SSE-ACC method is better than the AAC and *k*-mer methods. Moreover, it has a lower dimensionality. Therefore, we used the SSE-ACC features in the classification of effectors.

### Cross validation results

We conducted a 5-fold cross validation test on both the redundant (I) and non-redundant (II) data sets using LibSVM 2.82 [18] with an RBF kernel. The parameters used for these two data sets are *γ *= 0.25, *C *= 4, and *γ *= 0.5, *C *= 4. Table 2 lists the total accuracy, recall and precision of the predicted effectors on the two data sets, respectively.

**Table 2:** Cross validation results on the two data sets

Data	*TA*(%)	*P*(%)	*R*(%)
I	99.0	94.1	85.4
II	98.6	90.8	64.8

The experimental results show that while the total accuracy and precision of our prediction are high, the recall of the known effectors is relatively low (about 65% on the non-redundant data set). This is due to the class imbalance in the data sets. Since our goal is to find novel effectors, this prediction system (*i.e*., the trained SVM) with a low false positive rate could help reduce the cost of our future wet-bench experiments for validating the predicted effectors.

### Predicting type III secreted proteins in rhizobia

As an application of our method, we predicted T3SEs in rhizobia. The most significant characteristic of rhizobia is their ability to nodulate leguminous plants and fix atmospheric nitrogen. The type III secretion system has been shown to play an important role during the nodulation process of several rhizobial species. As multiple rhizobial species have the T3SS apparatus, the function and mechanism of T3SS in nodulation have received a lot of attention in the research field of plant-microbe interactions [19]. However, only a few rhizobial proteins have been confirmed to be secreted in a type III dependent manner (designated nodulation outer proteins or Nops). Therefore, computational tools are in great need to detect novel secreted proteins in rhizobia.

In rhizobia, T3SS is involved in establishing mutualistic associations, instead of pathogenic associations, with plant hosts. Although the biological effects of T3SS are different in rhizobia and *P. syringae*, they have similar secretion mechanisms. We checked the N-terminal sequences of some known rhizobial effectors in the literature, and found that they share many of the statistical biases found in the *P. syringae *effectors [15] (Table 3). Note that each of the known rhizobial effector possesses at least one feature (*i.e*., statistical bias), indicating that the secretion mechanisms are similar between *P. syringae *and rhizobia. Since the majority of the rhizobial effectors do not have all three features, we could not simply use them to predict type III effectors. Therefore, we took advantage of the large number of confirmed type III effectors in *P. syringae *and used them as the training data for the detection of rhizobial effectors. Four rhizobial strains that have been confirmed to possess T3SS were included in the test data. The following genomic sequences were analyzed:

**Table 3:** Presence of the statistical biases in confirmed type III effectors in rhizobia. Feature 1 means at least 10% Ser residues within the first 50 amino acids.

			Features		
Species	Effector	GI number	1	2	3
Sino	NopA	55668600	0	1	1
	NopP	63103266	1	0	0
	NolB	19749321	1	1	1
	NolX	52631913	1	0	1
	NopC*	255767012	1	1	1
	NopL*	2182720	1	1	0
	NopP*	2182742	1	0	0
	NopB*	2182730	1	1	1
	NopX*	2182728	1	0	1
Meso	NopB*	13475298	1	1	1
	NolX*	13475296	1	1	1
Brady	NodN*	27379070	0	1	0
	NolB*	27376923	1	0	1

Feature 2 means that an Ile, Leu, Val, or Pro is located at the third or fourth residue of the protein. Feature 3 means no Asp or Glu residues within the first 12 amino acids. The matrix is boolean, *i.e*., 1 means true and 0 means false. The involved rhizobial species are abbreviated as Sino for *Sinorhizobium*, Meso for *Mesorhizobium*, and Brady for *Bradyrhizobium*. The effectors marked by * are from the four strains considered in this study. The third column lists GI numbers from NCBI GenBank.

• *Sinorhizobium sp*. NGR234

- plasmid pNGR234a

• *Bradyrhizobium japonicum *USDA 110

-complete genome

• *Mesorhizobium loti *MAFF303099

-the chromosome and two plasmids

•*Sinorhizobium medicae *WSM419

- the chromosome and three plasmids

This test data set consists of a total of 22220 protein sequences. The detailed number of proteins in each strain can be found in Table 4.

**Table 4:** Number of sequences in the rhizobia data set and prediction results.

Strain	Original #	# Seq. with *tts *box	Predicted #	Unconfirmed #
WSM419	6213	160	9	9
MAFF303099	7272	142	12	8
USDA110	8317	279	30	23
NGR234	418	375	6	0
Total	22220	956	57	40

The strains are abbreviated as WSM419 for *Sinorhizobium medicae *WSM419, MAFF303099 for *Mesorhizobium loti *MAFF303099, USDA110 for *Bradyrhizobium japonicum *USDA110, and NGR234 for *Sinorhizobium sp*. NGR234. The original number means the number of proteins collected from the rhizobial strains. For MAFF303099 and NGR234, the numbers are the total numbers of proteins on both the chromosome and plasmids. The third column lists the numbers of sequences that have the *tts *motif in their promoters. The fourth column records the numbers of candidate effectors predicted by the SVM.

Before the prediction by the SVM, we performed a promoter search to screen the test data based on the following two considerations. (i) The consideration of promoters will increase the reliability of our prediction. (ii) Extracting the secondary structure and solvent accessibility information for over 20, 000 proteins in the test data is a computationally intensive work. As mentioned before, most secreted proteins have conserved promoter motifs (about 30 bps in length) in the upstream region of their encoding genes [9-12,16]. In rhizobia, the motif is called the *tts *box, which is the binding site of the transcriptional factor TtsI. This motif has been found in both *Sinorhizobium sp*. NGR234 and *Bradyrhizobium japonicum *[20,21], suggesting that it is conserved in multiple rhizobial species. We also found the conserved sequence pattern in the other two strains included in our test data set. Moreover, we have found TtsI homologs in the genomes of all four strains and they are highly conserved (data not shown).

We scanned the promoter regions of all test genes to filter out those that do not have the *tts *box. To do this, we adopted HMMER [22] to build an HMM profile according to the consensus sequence found in [20,21]. The model was then used to scan the promoter regions (up to 1000 nucleotides upstream of each start codon) of all test genes. The model returns an e-value for each sequence to indicate the likelihood of a *tts *box instance. Considering the divergence among the four strains, we set the e-value cut-off at a relatively high value, 10^-2^. A total of 956 proteins were found to have e-values lower than this cut-off.

We then performed the amino acid sequence-based prediction on these 956 proteins using the SVM trained on data set I. The SVM outputs prediction probabilities for both positive and negative classes on each test protein. Because the training data has a very imbalanced distribution (the negative class takes an overwhelming proportion) and the SVM has biased classification results, we lowered the cut-off of the probability for the positive class to 0.01. That is to say that, if a protein contains an occurrence of the *tts *box in its promoter region and it received a positive class probability of 0.01 or higher from the SVM, we regard it as a candidate of type III secreted proteins. The whole computation process took several ten hours. Most of the time was spent on extracting secondary structure and solvent accessibility information, which were computed by collecting multiple-alignment profiles found in public protein databases [23,24].

Using the above prediction procedure, we obtained 57 candidate effectors. Interestingly, 17 of these putative effectors have been verified as T3SEs by wet-bench experiments. For example, we predicted six candidate effectors, namely NopL, NopX, NopB, NopP, NopC, and Y4zC from the strain *Sinorhizobium sp*. NGR234, which all turned out to be true positives. Their detailed annotation can be found in NCBI GenBank [25]. Candidates predicted from the other rhizobial strains that are confirmed effectors are listed in Table 5. Interestingly, among the 13 effectors listed in Table 3, nine are from the four rhizobial strains in the test data set. Our prediction correctly identified eight of them except the NodN gene from *Bradyrhizobium japonicum *USDA 110 which is not in the test set of the SVM. (Its promoter has a weak *tts *box, but the strength of the signal did not pass the e-value cutoff that we chose in the promoter screen.)

To experimentally verify the predicted rhizobial type III effectors, we analyzed the gene *bll8244 *of *B. japonicum *USDA 110. We focused on this gene because our Mass Spectrometry data showed that a *bll8244 *homolog in *Sinorhizobium fredii *strain HH103 was secreted upon induction with genistein and in a type III dependent manner (Morgan and Ma, unpublished data). The genome of this strain is not fully sequenced; therefore it was not included in our bioinformatics analysis. The expression of rhizobial nodulation genes including the type III-related genes is induced by isoflanoids exudated from host legumes. For soybean microsymbionts like *Bradyrhizobium japonicum *and *Sinorhizobium fredii*, genistein is the plant signal inducing nodulation gene expression. A potential *tts *box was identified at -188 ~ -152 bp upstream from the start codon of *bll8244*. A DNA fragment spanning this promoter region and the N-terminal 200aa of Bll8244 was cloned into the plasmid vector pSP329. This truncated protein was also tagged with a hemagglutinin (HA) at the C-terminus to facilitate protein detection. *S. fredii *HH103 carrying the recombinant plasmid was grown in YEM medium with or without genistein. The expression and secretion of the truncated Bll8244 protein was then examined in the cell pellet and in the supernatant of the bacterial cultures respectively. Western blots showed that the truncated Bll8244 was only expressed and secreted in the presence of genistein, suggesting that Bll8244 is a type III-secreted effector.

**Table 5:** Experimentally confirmed secreted proteins in Bradyrhizobium japonicum USDA110 and Mesorhizobium loti MAFF303099.

Strain	Effector	Source
USDA110	nodulation protein NolB	NCBI GenBank
	bll1862	Ref. [21]
	blr1904	Ref. [21]
	blr2058	Ref. [21]
	blr2140	Ref. [21]
	bll8201	Ref. [21]
	bll8244	This study
MAFF303099	nodulation protein NolX	NCBI GenBank
	mlr8763 (*i.e*., NopB)	Ref. [30]
	mlr6361	Ref. [30]
	mlr6358	Ref. [30]

Table 6 presents predicted rhizobial T3SEs that have not yet been confirmed. Most of these putative effectors are hypothetical proteins with unknown functions. We are currently in the process of validating the above candidates using wet-bench experiments. Our results demonstrate that using the method developed in this paper, novel secreted proteins can be predicted effectively and efficiently. The method could be used to screen a whole bacterial genome for potential T3SEs within a day.

**Table 6:** Predicted secreted proteins in rhizobia that have not been confirmed experimentally.

Gene ID	Annotation	Position of *tts *box	Motif e-value	SVM probability
blr1704	hypothetical protein	-67 ~ -31	2.30E-06	0.92
bll1648	hypothetical protein	-260 ~ -224	9.60E-03	0.88
blr1854	hypothetical protein	-66 ~ -30	6.10E-07	0.86
mlr5875	hypothetical protein	-157 ~ -121	1.00E-02	0.86
mlr6331	hypothetical protein	-81 ~ -45	2.10E-03	0.69
Smed_1170	biotin-regulated protein	-107 ~ -71	8.30E-03	0.68
blr5999	hypothetical protein	-693 ~ -657	7.00E-03	0.67
bll1840	hypothetical protein	-74 ~ -38	5.60E-05	0.64
Smed_5711	hypothetical protein	-606 ~ -570	0.0047	0.55
bll1796	hypothetical protein	-930 ~ -894	1.40E-06	0.54
bll1804	hypothetical protein	-102 ~ -66	7.50E-10	0.51
bll8244	hypothetical protein	-188 ~ -152	9.80E-06	0.51
bll1636	hypothetical protein	-657 ~ -621	3.10E-03	0.50
Smed_4857	hypothetical protein	-826 ~ -790	0.0068	0.49
Smed_1856	putative signal peptide protein	-299 ~ -263	2.80E-03	0.48
Smed_4485	hypothetical protein	-637 ~ -601	0.005	0.4
bll0275	hypothetical protein	-395 ~ -361	8.50E-03	0.38
bsr1999	hypothetical protein	-264 ~ -227	4.20E-04	0.37
mlr3881	hypothetical protein	-483 ~ -447	9.80E-03	0.36
blr0325	hypothetical protein	-490 ~ -454	5.40E-03	0.35
mll5027	hypothetical protein	-377 ~ -340	9.20E-03	0.35
bll1848	hypothetical protein	-300 ~ -264	9.00E-08	0.34
bll5481	hypothetical protein	-128 ~ -92	6.50E-03	0.33
mlr0825	hypothetical protein	-535 ~ -499	5.70E-03	0.32
bsr8005	hypothetical protein	-89 ~ -53	5.60E-03	0.31
mlr1025	*	-764 ~ -728	8.60E-04	0.29
mlr7808	hypothetical protein	-906 ~ -869	6.70E-03	0.29
Smed_0887	hypothetical protein	-585 ~ -549	5.50E-03	0.27
blr6167	hypothetical protein	-250 ~ -214	9.50E-03	0.27
msl5783	hypothetical protein	-710 ~ -673	8.40E-03	0.25
Smed_1171	peptidase M23B	-993 ~ -957	8.30E-03	0.23
bll5622	hypothetical protein	-152 ~ -116	9.50E-03	0.19
bll1877	hypothetical protein	-101 ~ -65	1.80E-08	0.13
Smed_5269	hypothetical protein	-610 ~ -574	0.00014	0.12
blr1869	hypothetical protein	-147 ~ -111	3.50E-08	0.1
Smed_0286	hypothetical protein	-133 ~ -97	1.50E-04	0.09
blr0354	hypothetical protein	-482 ~ -446	5.80E-03	0.09
bll1810	hypothetical protein	-246 ~ -210	1.90E-07	0.08
bll1798	hypothetical protein	-90 ~ -54	1.40E-06	0.08
bll1797	hypothetical protein	-533 ~ -497	1.40E-06	0.04

Here, the position of the *tts *box in a promoter region is specified in terms of its distance from the respective start codon. The negative sign means that the promoter region is upstream of the start codon. The annotation * indicates a transcriptional regulatory protein that is also a nodulation competitiveness determinant. Genes that contain "bll" and "blr" in their IDs are from *Bradyrhizobium japonicum *USDA 110, genes that contain "mll" and "mlr" are from *Mesorhizobium loti *MAFF303099, and genes that contain "Smed" are from *Sinorhizobium medicae *WSM419.

### Comparison with the existing methods

We compared our method with two recently published methods for T3SS effector prediction, EffectiveT3 [26] and T3SS prediction [27], as well as with the AAC and *k*-mer methods for feature representation. Data set II (the non-redundant set) and all the verified rhizobial effectors were used to test the methods.

The method EffectiveT3 uses the naive Bayes algorithm as the classifier. The features used in the method include frequencies of amino acids and frequencies from two reduced alphabets, *i.e*., the 20 amino acids are condensed into reduced alphabets according to their biophysical properties and hydrophobic/hydrophilic characteristics. We selected its plant training set, which contains all effector sequences derived from *Pseudomonas syringae*, and used the default restriction value 0.95. Among the 108 positive samples and 3424 negtive samples, EffectiveT3 predicted 78 true positives and 357 false positives, resulting in a recall and precision of 72.2% and 17.9%, respectively.

The tool T3SS prediction uses a sliding-window technique and encodes each amino acid in a single window as a binary string of length 20. Either an artificial neural network (ANN) or an SVM can be selected as the classifier. The training set of this tool also contains effectors of *Pseudomonas syringae*. Here, we adopted ANN as recommended by the authors [27] and used the default threshold 0.4. The tool obtained 90 true positives and 285 false positives, yielding a recall of 83.3% and precision of 24%.

Both of the above recall values are high, but the precisions are pretty low. This is easy to explain. On one hand, the training sets used in these studies contain the known *Pseudomonas *effectors. On the other hand, the training sets have relatively balanced numbers of positive and negative samples. In the EffectiveT3 test, the negative set is twice as large as the positive set, while in the T3SS prediction test, the ratio is about 1:1. In our method, the ratio of effectors and non-effectors in the training data is close to their natural ratio in *Pseudomonas syringae*. This imbalanced training set helped us to obtain a high precision of 90.8%, while keeping the recall at 64.8%.

We also tested the tools for predicting new effectors from rhizobia, including 13 known effectors listed in Table 3 and 8 other verified effectors listed in Table 5. EffectiveT3 and T3SS prediction recognized 14 and 18 known effectors, respectively. Our method was also able to predict 18 of them.

In the experiments on the AAC and *k*-mer methods, we performed 5-fold cross validation study using these types of features as well using SSE-ACC features. We considered adopting single amino acid, di-mer and tri-mer compositions in the first 10, 20, 30, 50, and 100 N-terminal residues. The best result was obtained by using single amino acid composition in the first 100 residues with a total accuracy of 98.3%. The recall and precision of effectors are 55.6% and 84.5%, respectively, which are over 5% lower than those of the proposed SSE-ACC method.

## Conclusion

This paper introduces a machine learning method for predicting novel proteins secreted via the type III secretion system. To our knowledge, this is the first attempt to predict type III secreted proteins in rhizobia using a machine learning method. The method extracts features from N-terminal amino acid sequences and uses the SVM to classify the input features as secreted or non-secreted proteins. Computational experiments were conducted on *Pseudomonas syringae *and rhizobia data sets. The cross validation tests on the *P. syringae *data set showed that our method achieved a high accuracy and precision. With the optimum parameters found in the cross validation test, we trained the SVM classifier using a *P. syringae *data set, and tested the classifier on the rhizobia data set. In order to increase the reliability of our prediction, we screened the rhizobia data and removed proteins that do not have the *tts *box in their respective promoters. Our prediction resulted in 57 novel secreted proteins in the four rhizobial strains, among which, 17 have been confirmed as true positives.

This new computational method will contribute to the identification of novel type III secreted effectors and advance our understanding on type III secretion mechanisms. A better understanding of the molecular mechanisms underlying type III secretion will contribute to the development of novel strategies for controlling bacterial diseases and promoting yields in agriculture.

## Methods

### Classification system building

The classifier is built using the state-of-the-art supervised learning machinery, the SVM, which is widely used in bioinformatics. Our implementation of the SVM adopted LibSVM version 2.8 [18]. We considered polynomial, sigmoid and RBF kernels for the SVM, and observed that the RBF kernel has the best classification accuracy.

Each feature vectors consists of two parts, the amino acid composition on three secondary structural elements and the amino acid composition on two solvent accessibility states. The secondary structure elements were predicted by PSIPRED [23], and the solvent accessibility states were predicted by ACCpro [24]. Both of them are highly accurate prediction methods. All the feature vectors were scaled in the range of [0, 1] using SVM-Scale in the LibSVM package [18].

### HMM construction

An HMM profile was built on the promoter patterns (*i.e*., the *tts *box) extracted from *Sinorhizobium *sp. NGR234 and *Bradyrhizobium japonicum*. Marie *et al*. [20] gave an alignment of 11 *tts *boxes identified on the symbiotic plasmid of NGR234, and summarized a consensus sequence as tcGTCAGcttntcGaaAGctnngccncnta. In the consensus sequence, highly conserved nucleotide positions (*i.e*., with frequencies ≥90%) are shown in uppercase letters. Lowercase letters are used for nucleotides conserved in at least 50% of the sequences, and n means any nucleotide. Recently, Zehner *et al*. searched *tts *box motifs in the genome of *Bradyrhizobium japonicum*, and summarized a similar consensus sequence pattern: tcGTCAGcTtntcGacAGctagnccnnntA [21]. Note that these two consensus patterns are very similar, especially on the highly conserved positions.

We collected the *tts *box sequences from both above rhizobial species, aligned them by using ClustalW [28], and then used HMMER [22] to build an HMM profile to represent the *tts *box.

### Protein secretion assay

The DNA sequence carrying the predicted *tts *box (-200 bp upstream of the start codon of *bll8244 *gene) and the first 200aa of Bll8244 was cloned in the plasmid vector pSP329. In order to facilitate protein detection in the supernatant of liquid culture, this partial protein was in-frame fused to a hemagglutinin (HA) tag at the C-terminus. The recombinant plasmid was then introduced into *Sinorhizobium fredii *HH103 by triparental mating. *S. fredii *HH103 carrying pSP329::*bll8244*-HA was grown in YEM medium [29] supplemented with tetracycline (2.5 *μ*g/mL) at 28°C for 1-2 days. This culture was used as a preculture to reinoculate YEM medium at an OD600 = 0.5. The cells were induced with genistein (1 *μ*g/mL) for 48 hours before the cell-free supernatant was collected by multiple centrifugations in order to completely get rid of the cells in the liquid culture. The proteins in the supernatant were precipitated according to Vinardell *et al*. [29]. Protein pellets were resuspended in 2 × Laemmli buffer and analyzed using SDS-PAGE. The expression and secretion of truncated Bll8244 was detected by western blots using anti-HA antibody.

## Competing interests

The authors declare that they have no competing interests.

## Authors' contributions

Y. Yang and W. Ma designed the system. Y. Yang performed the computational tasks. J. Zhao, R.L. Morgan and W. Ma conducted wet-bench experiments. T. Jiang supervised the project. All authors read and approved the final manuscript.
